# Bone Response to Surface-Modified Titanium Implants: Studies on the Early Tissue Response to Implants with Different Surface Characteristics

**DOI:** 10.1155/2013/412482

**Published:** 2013-09-23

**Authors:** C. Larsson Wexell, P. Thomsen, B.-O. Aronsson, P. Tengvall, M. Rodahl, J. Lausmaa, B. Kasemo, L. E. Ericson

**Affiliations:** ^1^Institute of Anatomy and Cell Biology, University of Göteborg, Göteborg, Sweden; ^2^Department of Oral and Maxillofacial Surgery, SÄS, 501 82 Borås, Sweden; ^3^Department of Biomaterials, Sahlgrenska Academy at University of Gothenburg, Göteborg, Sweden; ^4^BIOMATCELL VINN Excellence Center of Biomaterials and Cell Therapy, Göteborg, Sweden; ^5^Department of Applied Physics, Chalmers University of Technology, Göteborg, Sweden; ^6^Nano Bridging Molecules SA, 1196 Gland, Switzerland; ^7^Department of Physics and Measurement Technology, Linköping University, Linköping, Sweden; ^8^Department of Chemistry, Materials and Surfaces, SP Technical Research Institute of Sweden, 501 15 Borås, Sweden

## Abstract

In a series of experimental studies, the bone formation around systematically modified titanium implants is analyzed. In the present study, three different surface modifications were prepared and evaluated. Glow-discharge cleaning and oxidizing resulted in a highly stoichiometric TiO_2_ surface, while a glow-discharge treatment in nitrogen gas resulted in implants with essentially a surface of titanium nitride, covered with a very thin titanium oxide. Finally, hydrogen peroxide treatment of implants resulted in an almost stoichiometric TiO_2_, rich in hydroxyl groups on the surface. Machined commercially pure titanium implants served as controls. Scanning Auger Electron Spectroscopy, Scanning Electron Microscopy, and Atomic Force Microscopy revealed no significant differences in oxide thickness or surface roughness parameters, but differences in the surface chemical composition and apparent topography were observed. After surface preparation, the implants were inserted in cortical bone of rabbits and evaluated after 1, 3, and 6 weeks. Light microscopic evaluation of the tissue response showed that all implants were in contact with bone and had a large proportion of newly formed bone within the threads after 6 weeks. There were no morphological differences between the four groups. Our study shows that a high degree of bone contact and bone formation can be achieved with titanium implants of different surface composition and topography.

## 1. Introduction

This study is part of a multidisciplinary approach where the long-term objective is to understand the role of specific surface properties when bone and marrow are exposed to an implant. The objective and rationale for the approach are presented in an earlier report [1]. In short, the surfaces of machined, threaded titanium implants are modified and characterized in different ways and the bone response and bone-implant interface are investigated *in vivo* [1–3]. Whereas earlier studies addressed the role of roughness and surface oxide thickness, modified by electrochemical methods, in this study we inquire further into smaller chemical changes on implant surfaces. The hypothesis is that surface chemical composition does influence the tissue response including the bone response to titanium implant. We chose machined titanium implants as the control because this was the starting material for three different surface modifications that were studied.

In previous studies, we examined the response of bone around threaded titanium implants with different surface modifications (machined, electropolished, machined, and electropolished with different oxide thicknesses obtained by anodic oxidation). The formation of bone was evaluated morphometrically after 1, 3, 6, 7, 12, and 52 weeks. At the earlier time periods, the formation of bone was less around especially the very smooth electropolished implants. The results suggested that the type of surface modification performed mainly influenced the early phase of bone regeneration around the implants. In the present study, we have examined the biological response of cortical bone around titanium implants modified with respect to both surface structure and chemical composition by glow-discharge plasma techniques and by hydrogen peroxide treatment, 1, 3, and 6 weeks after implantation.

At first, the implants were electropolished since morphology and microstructure of the surface oxides depend strongly on the microstructure of the underlying metal over which the oxide grows. Machined and mechanically polished surfaces consist of a plastically deformed, amorphous layer which may extend several microns into the bulk of the material. Electropolishing or other etching treatments remove this amorphous surface layer, resulting in surfaces that have a polycrystalline termination in which the grain structure of the material is visible. The thinnest thermal oxides on titanium have a very homogenous and essentially featureless morphology.

Glow-discharge techniques are widely used for cleaning, sterilizing, and modification of biomaterial surfaces [1, 4–9]. These methods offer great advantages with respect to the possible range of modifications, as well as to process control (purity). With appropriate plasma parameters, argon plasmas remove all chemical traces at the surface from former treatments, such as adsorbed contaminants, impurities, and native oxide layers [4]. On such a “cleaned” metallic surface, new surface layers can thereafter be built up under well-controlled conditions in the vacuum chamber of the plasma equipment. It is further possible to perform *in situ* characterization of the resulting surface properties. 

There are different methods to grow surface oxides on metals in a controlled way, electrochemically or by glow discharge plasmas. Other techniques used are, for example, physical vapor deposition (PVD) techniques and thermal oxidation. The anodic oxidation and discharge (DC) plasma oxidation techniques enhance the diffusion rate of oxygen and titanium atoms and make it possible to grow thick oxides on metals [4]. With glow-discharge plasmas it is possible to both prepare and then characterize the obtained surface in ultrahigh vacuum equipment, which gives a better control over the preparation procedure. Glow-discharge plasma treatment has early on been proposed as a cleaning and sterilization method for metal instruments [10–12]. Glow-discharge treatment generally results in high surface energy [13–15] immediately after the treatment, due to the removal of material and creation of unsaturated surface bonds. This in turn makes the surface reactive and prone to rapid oxidation and recontamination due to reaction with oxygen and adsorption of airborne contaminants such as hydrocarbons. The samples in this study were exposed to pure oxygen or pure nitrogen prior to air exposure, thereby reducing the amount of contaminants from the air. Substituting the pure oxygen with nitrogen makes it possible to form TiN in a controlled way. Dion and coworkers [16, 17] have shown *ex vivo* and with a physical vapor deposition (PVD) technique on Ti-alloy that compared with silicon, TiN surfaces do not adsorb as much albumin/fibrinogen as silicon surfaces do. However, the protein layer on TiN (fibril meshwork) entrapped mainly red cells and no leukocytes compared with silicon adsorbing platelets retaining red blood cells and a large amount of leukocytes. N-ion implantation is another surface treatment technique used with the purpose of increasing the protection against ion release (corrosion) and eventually increases biocompatibility [18, 19]. 

The third surface modification method used is based on previous studies [20–23] on the interaction between hydrogen peroxide, that is formed during inflammatory conditions and titanium. Hydrogen peroxide, H_2_O_2_, is a strong oxidizer and is also used in cleaning solutions [24]. *In vitro* studies of the interaction between metallic titanium and more concentrated H_2_O_2_ have shown that titanium peroxide (TiO_2_^2^-) and titanium superoxide (TiO_2_-) were formed [21–23].

In the present study, we have used glow-discharge plasma procedures, developed in our laboratory [4] for preparing clean oxide surfaces and nitride layers and a process of hydrogen peroxide treatment of titanium implants.

Reported results show that neither of these, relatively dramatic, surface modifications lead to significant differences in early bone healing around the titanium implants in this model. In the present study, we have examined the biological response in cortical bone 1, 3, and 6 weeks after implantation of titanium implants modified by changing the surface characteristics (Table 1).

## 2. Materials and Methods

### 2.1. Implant Preparation

Ninety-six threaded implants (*∅*3.75 mm, length 4.0 mm) were manufactured by machining from a commercially pure (99.7%) titanium rod. They were divided into four different groups. The preparation procedures are briefly summarized in Table 1 together with the resulting surface characteristics.

After machining and before any further treatment, all samples (including controls) were ultrasonically cleaned in successive baths of trichloroethylene, acetone, and ethanol of analysis grade purity for ~15 minutes in each.

Two groups (24 + 24 samples) of machined implants were modified using a glow-discharge procedure, carried out in different gases and resulting in the formation of two different surface compositions, titanium oxide and titanium nitride layers, respectively. The glow discharge method has been described in detail elsewhere, and it will only be briefly described here [5]. The implants were treated in a plasma treatment chamber, built in-house. A DC discharge (2.0 kV) in pure Ar-gas (99.9990% at 30 Pa) was applied for 10 minutes between the sample acting as the cathode and a concentrically placed cylindrical anode. The treatment was carried out in UHV equipment with cylindrical electrode geometry. This procedure has been shown to be highly efficient in cleaning the surface and capable of complete removal of surface contaminants on the native oxide layers on screw-shaped Ti samples [4]. After this cleaning step, one group of samples was immediately reoxidized *in situ* in pure oxygen (99.998%, 1000 hPa) for 5 minutes in order to form a pure surface oxide consisting of titanium dioxide (TiO_2_). The other group was subjected to glow-discharge plasma (2.0 kV) in pure nitrogen gas (99.9990%, at 40 Pa) for 5 minutes to form a thin titanium nitride film.

The fourth group (24 samples) was, after the ultrasonic cleaning procedure, incubated in a 10 mM H_2_O_2_ (Merck, 30%) solution for 40 hours at 8°C, in order to form a sterile, peroxidized surface [21–23]. After the preparation, the control and the plasma prepared samples were placed in specially designed, cleaned, and sealed closed titanium cylinders serving as separate containers for each implant. The cylinders were then put in polymer sterilizing bags, taped, and *γ*-irradiated at 28.9 kGy for 25 h at 30°C. Bacteriological and sterile tests were proved negative for all four sample groups.

### 2.2. Implant Characterization

#### 2.2.1. Chemical Composition

The surface elemental composition of two samples of each preparation type was analyzed with Scanning Auger Electron Spectroscopy (AES), (Perkin Elmer PHI660, Eden Prairie, USA). Survey spectra (30–1730 eV) in two or five points located in the thread portion of each implant and depth profiles in one or two points at each sample were recorded. All spectra were taken at 5 keV primary electron energy with an e-beam current of 1.5 *μ*A, e-beam diameter of 180 *μ*m, and energy resolution of 0.6%. Relative concentrations (in atomic percent) of the detected elements were calculated from their peak-to-peak values in differentiated spectra after correction with the elemental sensitivity factors [25]. This procedure gives the average concentrations of the detected elements within the probed volume (typically the 3–10 outermost atomic layers) and does take into account neither the depth distribution of the elements, nor chemically induced variations in the sensitivity factors. Therefore, the quoted concentrations should not be regarded as absolute surface concentrations. However, comparison between the different samples can be made, since they were analyzed under identical conditions. Oxide thickness was estimated from AES depth profile analysis using 2 keV Ar-ions for sputtering. The oxide thickness was taken as the depth at which the oxygen signal had decreased to half of its intensity at the oxide surface. The sputtering rate, as calibrated for Ta_2_O_5_, was 5.2 nm/min, which corresponds to approximately 2.6 nm/min for TiO_2_.

Since the Auger electron signals from Ti and N overlap, X-ray photoelectron spectroscopy (XPS) was also used for a more accurate determination of the chemical composition of the nitride sample. Both survey spectra (0–1100 eV binding energy) and high-resolution spectra of the Ti 2p, N 1s and O 1s peaks were recorded, using the standard Mg-*α* source and a monochromated Al-*α* source, respectively, of a PHI 5500-system (Perkin-Elmer, USA).

#### 2.2.2. Surface Topography and Roughness

Scanning Electron Microscopy (SEM, Zeiss DSM 982 Gemini, Germany) was used to obtain an overall picture of the surface topography of the samples. SEM micrographs were taken at several randomly chosen areas on the implant surfaces. 

A quantitative characterization of the nanoscale surface topography and roughness was carried out by Atomic Force Microscopy (AFM, Nanoscope III, Digital Instruments, USA). Standard Si_3_N_4_ tips were used for imaging in the contact mode. One sample of each preparation type was analyzed at ten randomly chosen areas, (1 × 1 *μ*m^2^ and 256 × 256 pixels) on the flat part at the bottom of the implant. This location on the implant was shown in a previous study to give essentially the same result as analysis on the threaded part [3].

The surface roughness (*R*_rms_) of each imaged area was quantitatively evaluated using the computer software of the AFM instrument, and mean values were calculated for each type of surface. In addition, the AFM images were also used to calculate the surface area enlargement (*A*_diff_). This parameter represents the enlargement in surface area (in percent of the projected area) caused by surface roughness in the range from a few nm (resolution of the images) up to 1 *μ*m (size of the imaged area). The surface area enlargement was estimated from the sum of the area of all triangles formed by three adjacent pixels divided by the projected image area [26]. Additional topographical characterization on the micro-scale (*R*_*a*_-value) was obtained by an optical profilometer for three-dimensional measurements, TopScan3D (Heidelberg Instruments GmbH, Germany) [27].

### 2.3. Animals and Surgery

Twenty-four adult New Zealand white female rabbits, weighing 3-4 kg, were used. The experiments were approved by the Local Ethics Committee. The animals were allowed to run free in a specially designed room with food and water ad libitum. The procedures for surgery and implant insertion are described in detail in previous reports [1, 3]. In summary, a standard procedure for implant installation was carried out with careful surgical technique, generous irrigation with saline, and low-speed drill (2000 rpm). After prethreading, two implants were inserted 10 mm apart in each proximal tibial metaphysis in a pre-determined order; thus, each animal received one implant of each type.

The animals were sacrificed with an overdose of barbiturates intravenously and fixed by perfusion with 2.5% glutaraldehyde in 0.05 M sodium cacodylate buffer, pH 7.2. The implants and surrounding tissue were removed *en bloc*, further immersed in glutaraldehyde overnight and then postfixed in osmium tetroxide for two hours. After dehydration, the undecalcified specimens were embedded in plastic resin, L R White (The London Resin Co. Ltd., Hampshire, England).

### 2.4. Morphology and Morphometry

Ground sections of 10–15 *μ*m thickness were prepared [28] and examined, using Leitz Microvid equipment connected to a personal computer. Measurements were performed directly in the microscope. The contact ratio between the implant surface and bone tissue was calculated. Similarly, the proportion of bone tissue within the threads along the implant was calculated. The data are given as percentage bone-implant contact (referred to as bone contact) and percentage of the total area within the threads containing mineralized bone (referred to as bone area). All five consecutive threads (with number 1 and 2 located in the cortex) were evaluated. The mean of the left and right sides of the section and mean values for each thread in the different groups were calculated.

### 2.5. Statistics

The Fisher exact test for paired samples was used.

## 3. Results 

### 3.1. Implant Surface Characterization

#### 3.1.1. Surface Composition and Oxide/Nitride Thickness

The relative concentrations (in atomic %) of the detected elements present on the sample surfaces, as measured by AES, are presented in Table 1. On all samples the dominant peaks were from Ti, O or N/O, and C. All samples, showed carbon levels of 10–15 at %, which is low in comparison with other studies (typically 30% or more) [29]. The shapes of the TiLMV peaks indicated that the oxides on the control, the glow discharge oxidized, and the H_2_O_2_ incubated samples, respectively, were nearly stoichiometric titanium dioxide. The depth profiles also showed a similar oxide thickness (2-3 nm) for these three groups. 

Glow-discharge plasma treatment in pure nitrogen resulted in 3 nm thick stoichiometric titanium nitride films, as judged from the depth profiles for the TiLMM + NKVV and TiLMV peaks, respectively [30–33]. The presence of titanium nitride was also evident in the XPS spectra, which showed N 1s and Ti 2p peak positions and shapes consistent with Ti nitride. The oxygen detected on the nitride samples was shown to be present only on the outermost surface. The binding energy of the XPS O 1s signal indicated that most if not all of the oxygen was bound to carbon, that is, in organic molecules adsorbed on the very surface from air exposure. However, the formation of a small amount of titanium oxide (or oxygen dissolved in the nitride) cannot be excluded [34–36]. The presence of large amounts of nitrogen in the form of a titanium nitride constitutes a markedly different surface chemistry compared to the other groups. 

Further, when comparing the preparations made in our earlier studies [1–3], it can be concluded that overall cleaner implant surfaces were obtained when using well-controlled glow-discharge plasma treatments and *γ*-irradiation rather than wet chemical procedures and autoclaving.

#### 3.1.2. Surface Topography and Roughness

**Figure 1:**
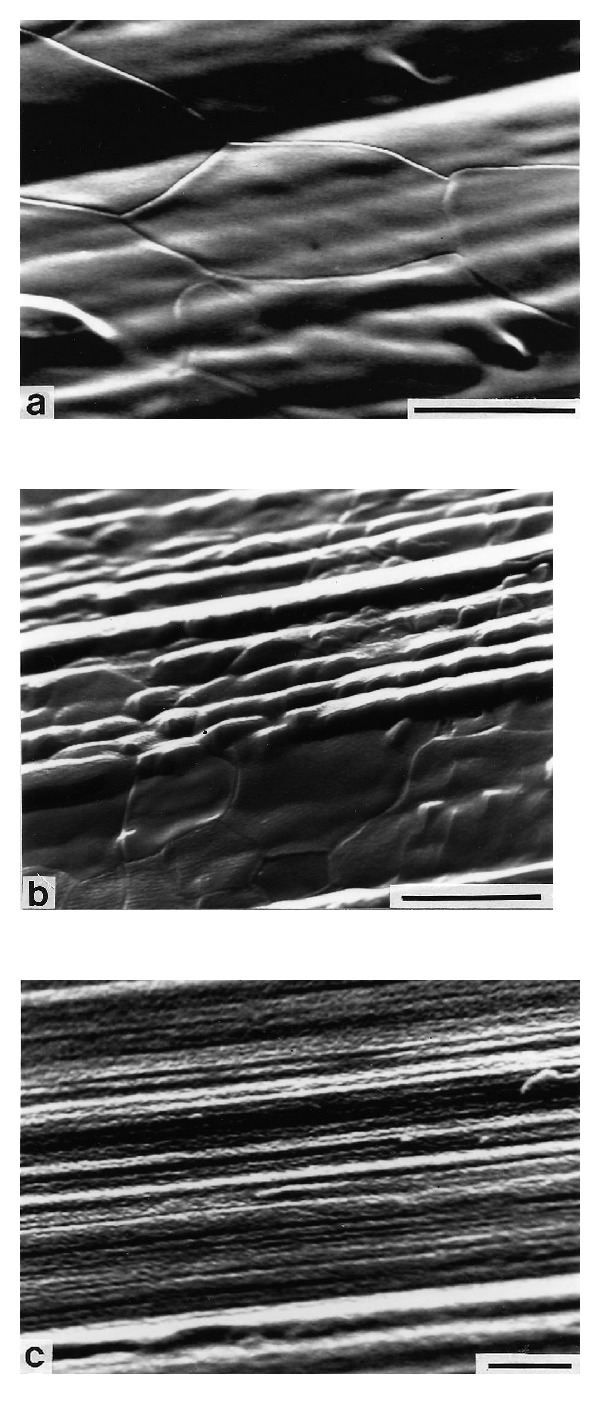
SEM images showing the surface topography of the samples (a) glow discharge cleaned and thermally oxidized sample. Bar = 5 *μ*m; (b) glow discharge cleaned and nitrided sample. Bar = 5 *μ*m; (c) hydrogen peroxide treated sample. Bar = 2 *μ*m.

**Figure 2:**
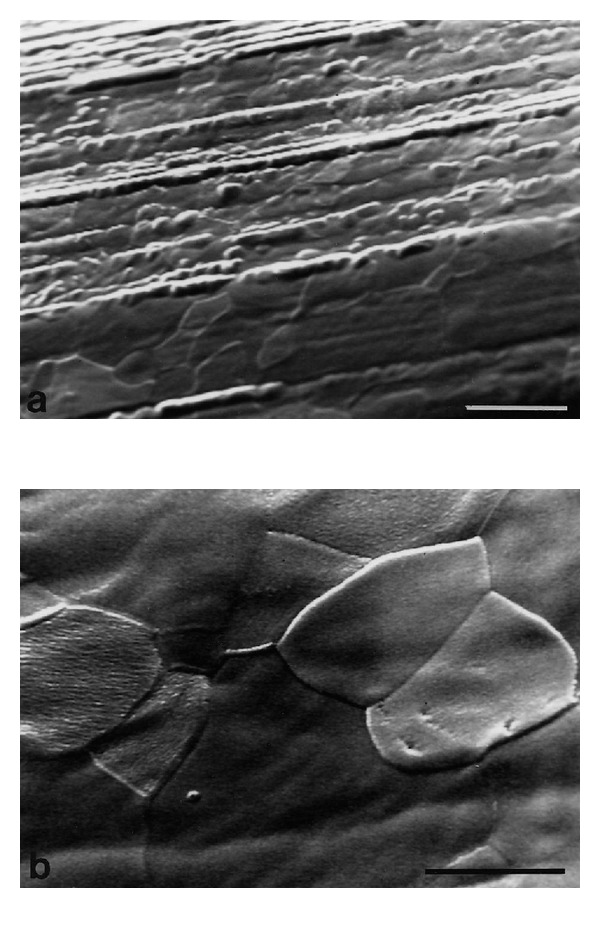
SEM images of the glow-discharge nitrided sample; (a) the machining grooves are smoothened and grain structures are visible. Bar = 10 *μ*m; (b) the surface topography of the individual grains is relatively smooth. Bar = 5 *μ*m.

Figures 1 and 2 show representative SEM images of plasma treated and H_2_O_2_ incubated samples. The quantitative AFM results are given in Table 2. The control sample (SEM and AFM image not shown) had the typical topography of machined samples characterized by machining grooves (on the scale up to 10 *μ*m) which are oriented in the cutting direction. The surface roughness parameters, *R*_rms_, as measured by AFM and optical profilometry were 26 nm and 0.6 *μ*m, respectively, and in agreement with previous work. The seemingly large difference of these results stems from the difference in measured topographical features between the two methods. AFM gives information on a nanometer scale (*z*-range: 1 nm–6 *μ*m; lateral range: 1 nm–100 *μ*m), while the optical technique gives information on a micrometer scale (*z*-range: 6 nm–108 *μ*m; lateral range: 1 *μ*m–2 mm).

The two plasma treated samples (Figures 1(a), 1(b), and 2) have qualitative similar surface topographies which, however, are distinctly different from the other two sample groups. The surfaces have a relatively smooth appearance in the SEM, with clearly visible grains and grain boundaries. While the majority of the grains have smooth surfaces, some of them show a corrugated topography on the submicron level, both of which are characteristics of sputter-etched surfaces. The AFM analysis revealed a wavy structure with amplitude of about 50 nm and a period of 10 nm at the nitrided sample. In contrast to the oxidized samples which were only reoxidized in pure oxygen after the argon plasma cleaning step, the nitrided group was subjected to a second plasma treatment in N_2_, which presumably leads to the observed differences in topography. Quantitatively, the two plasma treated surfaces differ somewhat: the oxide sample has a lower roughness, while for the nitride it is similar to the control sample. At the submicron level, the plasma oxidized samples had a lower *R*_rms_ value than the other implant surfaces. The *R*_*a*_-values were within the range of 0.4–0.7 *μ*m. The nitrided sample, however, has a larger surface enlargement than the oxidized surface (Table 2).

The H_2_O_2_ treated sample (Figure 1(c)) shows clear traces from the machining. In addition, an irregular roughness on the submicron level from etching in H_2_O_2_ is superimposed on this topography. The roughness of this sample is similar to the control samples, but with a larger surface area (Table 2). This topography reflects the etching action of the peroxide treatment.

### 3.2. Bone Morphology and Morphometry

**Figure 3:**
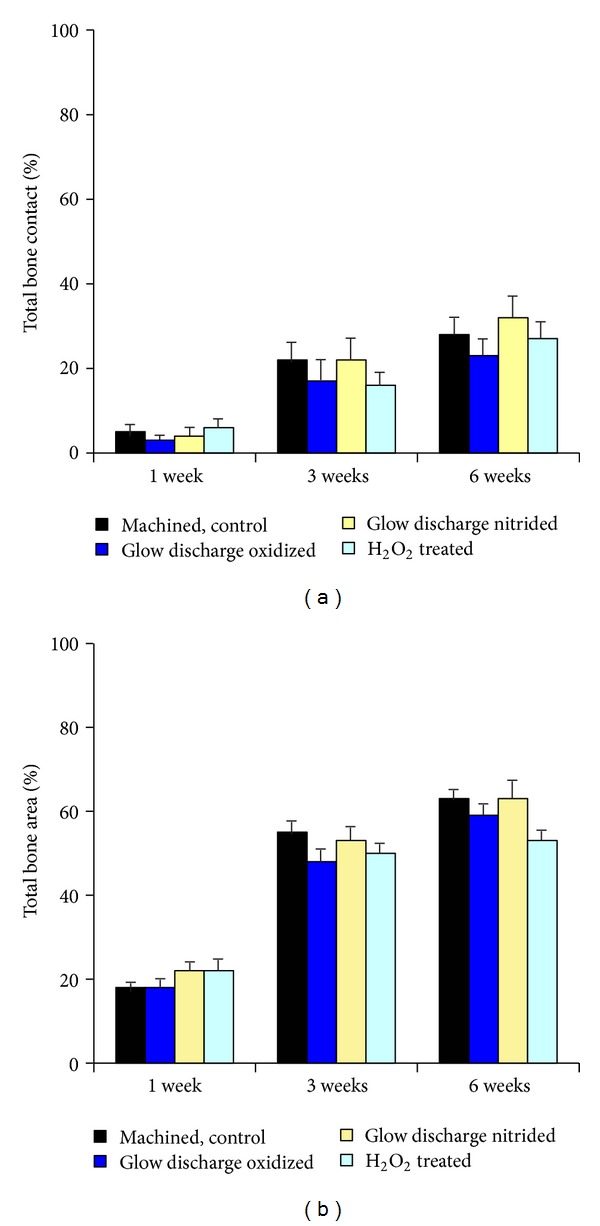
Morphometry. (a) Total bone contact (%) after 1, 3, and 6 weeks. Mean + s. e. (b) Total bone area (%) after 1, 3, and 6 weeks. Mean + s. e.

The results of the morphometric evaluation of the relative bone area and bone-implant contact for the entire implant are shown in Figures 3(a) and 3(b). No significant differences were observed between the mean values of the different groups.

**Figure 4:**
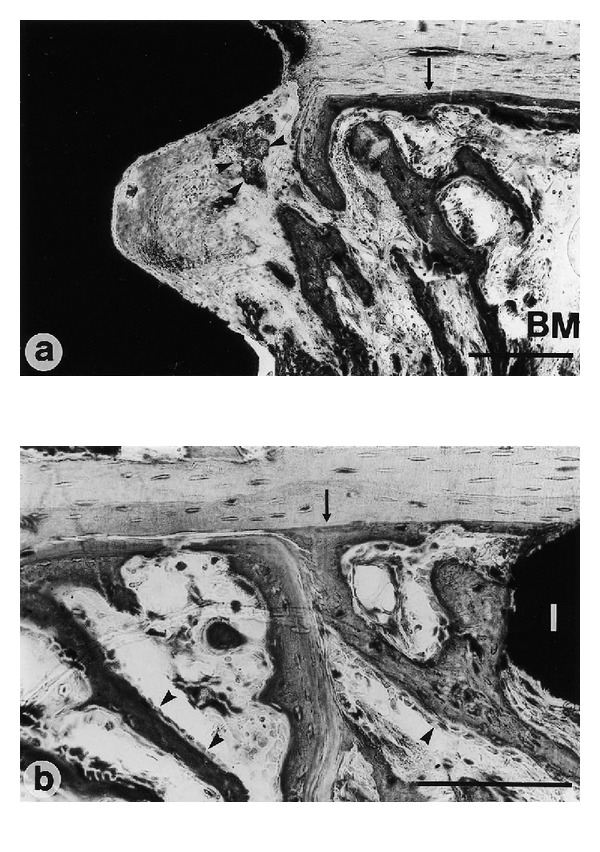
((a), (b)) Solitary osteoid formation is found within the threads of the implant (arrow-heads). (a) Endosteal trabeculae in the bone marrow (BM) are protruding towards the implant surface. The border between the “old” cortex and the newly formed bone (arrows) is clearly visible. Bar = 200 *μ*m. (a) Machined (control) sample and (b) glow discharge cleaned and thermally oxidized sample. one week after implantation. The osteoblasts are lining the newly formed trabeculae (arrow heads). Bar = 200 *μ*m.

**Figure 5:**
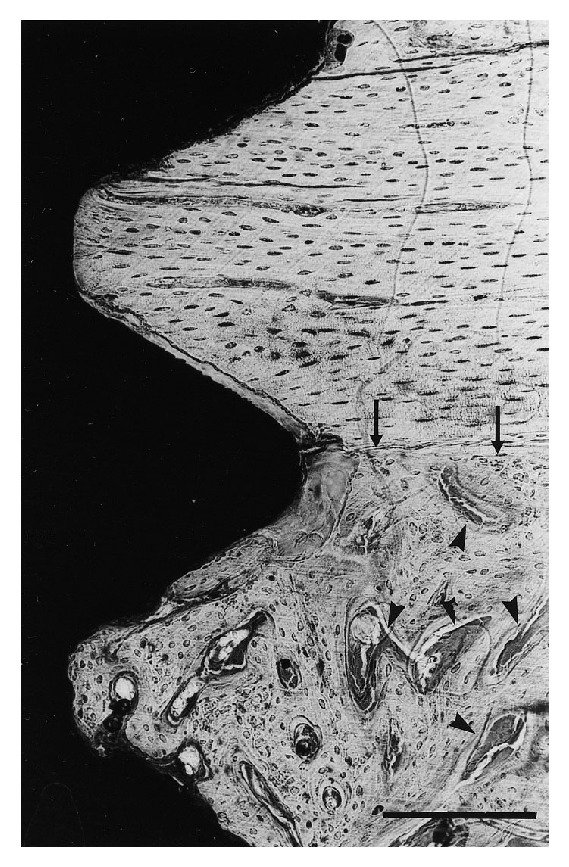
H_2_O_2_ treated implant after 1 week. An intense remodeling activity (arrow-heads) is observed immediately located beneath the corticomedullary border (arrows). Bar = 200 *μ*m.

One week after implantation, formation of new bone was observed as trabecular woven bone covered with osteoblast seams at the endosteal surface, beginning 1–1.5 mm from the implant surface. At this time period, solitary osteoid formations were detected within the threads of the implant. In addition, long bone trabeculae reached down from the endosteal surface in the bone marrow towards the implant (Figures 4(a) and 4(b)). No bone formation was seen at the cut edge (drilling hole) of the cortex. A large amount of woven bone filled the threads located in the original cortex and the bone was to a large extent in contact with the surface of the implant (Figure 5). Only about 5% of the implant surface was in direct contact with mineralized bone after 1 week, and no quantitative differences were found between the different groups (Figures 3(a) and 3(b)).

**Figure 6:**
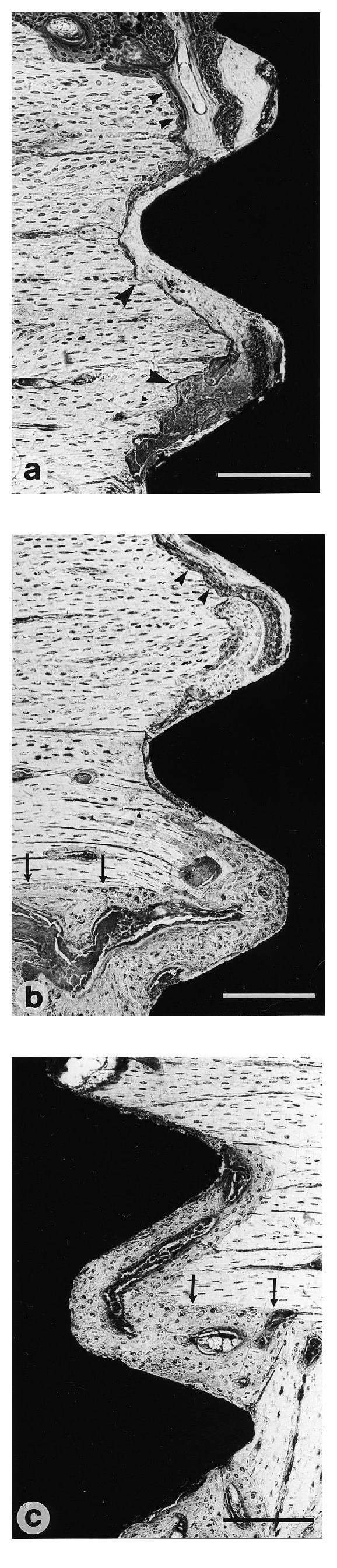
Three weeks after implantation. The cortical bone surface facing the implant surface is characterized by marked signs of resorption (large arrow heads) and the occurrence of new bone formation (small arrow heads). The border between the lamellar cortical bone and the newly formed bone is visible (arrows); (a) Machined (control) sample. Bar = 200 *μ*m; (b) glow discharge cleaned and nitrided sample. Bar = 200 *μ*m; (c) glow discharge cleaned and thermally oxidized sample. Bar = 200 *μ*m.

Three weeks after implantation, resorption was clearly observed on the cortical surface close to the implant surface (Figures 6(a)–6(c)). All threads, including the threads located in the bone marrow, contained a large amount of newly formed woven bones.

**Figure 7:**
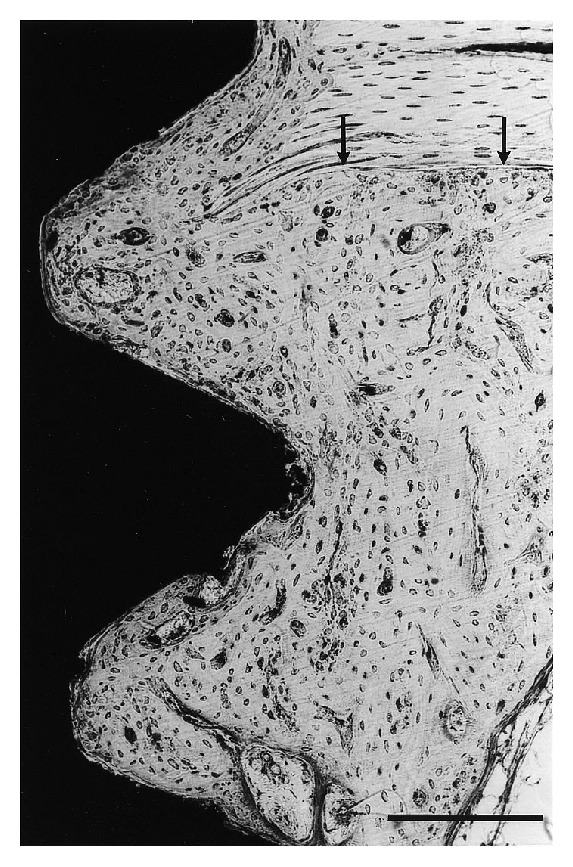
Six weeks after implantation. The implant surface towards the marrow cavity was in general in continuity with the cortical bone. Close bone-implant contact was seen for all implant types, and the threads were filled with woven bone. H_2_O_2_ treated implant. Bar = 200 *μ*m.

After 6 weeks, about 80% of the area within threads located in the original cortex (threads 1–3) were filled with bone. There were no qualitative differences between the different implant types. The implant surface towards the marrow cavity was covered by a layer of bone which was in continuity with the cortex. This newly formed bone had commonly a woven character (Figure 7). The parts of the implant surface which were in contact with soft tissue, contained blood vessels, mesenchymal cells, and occasional multinuclear cells. Osteoblasts or osteoid seams were rarely seen in direct contact with the implant surface. 

The sequence of bone formation around the implants essentially followed the same pattern as that previously described for machined, electropolished, and anodized titanium implants in the cortical bone of rabbits [3, 37] as well as for rats [38]. Further, the increase of bone contact and bone area parameters is consistent with these from previous kinetic studies.

## 4. Discussion

The biocompatibility and kinetics of the bone formation process, may be either enhanced or suppressed by the surface properties. Excessive release of metal ions from the material may be one such potentially suppressing effect. From a biological point of view, strategies may therefore be to optimize the surface properties in order to reduce the negative effects [9, 39]. Both a TiN coating and a thick TiO_2_ decrease the diffusion of Ti ions from the bulk metal, which may be of potential interest when implanted *in vivo* [40, 41]. 

In the present study, the bone response to the TiN implants did not differ significantly from that to the other implanted materials, including the machined implant with a native TiO_2_. In the literature, there are several reports on the biological reactions at TiN surfaces, mainly prepared by PVD and CVD techniques, including the responses of bone [42–44], soft tissues [42, 45] blood [16, 17, 46, 47], platelets [48], human mesenchymal stem cells [49], and osteoblasts [50, 51]. Several of those studies indicate that TiN surfaces have beneficial or comparable properties in comparison with other currently and frequently used materials.

Another strategy is to chemically modify the titanium and titanium oxide surfaces by incorporation of, for example, cations such as lanthanum [52]. At physiological pH the hydrated TiO_2_ has a net negative surface charge, thus attracting cations like calcium, [53]. Chemical treatment of TiO_2_ powder (anatase) by adsorption of lanthanum cations resulted in an increased adsorption of albumin and serum proteins in comparison with controls [52]. Furthermore, 2–10 weeks after implantation in rats and rabbits of lanthanum treated titanium implants, a fibrous encapsulation and lower push-out values than controls were recorded. On the other hand, pretreatment with fluoride ions was shown to increase the push-out values [54] and bone morphometric values [55].

These and other observations indicate that the surface charge influences the bone tissue response, possibly by influencing the types and amount of proteins that are adsorbed to the surface. The interactions between proteins, cells and implant surfaces may be influenced not only by the chemical properties of the surface but also the surface roughness which in turn may influence the wettability (hydrophilicity) which play an important role. [56]. Therefore, optimization of both surface chemical and topographical properties need to be considered when new materials are designed [57]. The role of implant surface energy and cleanliness for interfacial events, molecular adsorption, and cellular adhesion has been addressed by several authors [7, 14, 15, 57–62].

*In vitro* studies on osteoblast-like cells [63, 64] and osteoclasts have been performed on glow-discharge titanium [65] and titanium alloy plates [66]. Increased platelet adhesion and activity [66], as well as increased protein adsorption [63, 65] are factors that are demonstrated to influence the cellular response on surfaces with high wettability. In addition, results from recent *in vivo* studies indicate enhanced bone response on titanium surfaces with higher surface energy [60, 67, 68] under experimental conditions. 

The surfaces subjected to the present preparation techniques followed by sterilization using *γ*-irradiation and subsequent air exposure had a relatively low amount of surface contaminants. Previous studies have shown that machined and electropolished titanium implants with and without thick (180–220 nm) oxides have hydrophilic surfaces (water contact angles 15–33 degrees) with the highest contact angles observed for electropolished and machined surfaces [69]. In the latter study, oxide thickness and carbon contamination had no clear influence on protein adsorption and activation of blood coagulation [69]. In the present study, the amount of carbon contamination was lower than that detected on our previous samples [1, 3]. However, comparisons between samples analyzed at different occasions should be made with caution. Interestingly, in comparison with the earlier bone morphometry data, the present degree of bone-implant contact and amount of bone within threads were higher. These observations indicate that surface contamination may be one of several important factors influencing the biological response. This has also been observed by Aita et al. [70] who investigated the effect of UV irradiation on the biological response to titanium surfaces. In that study, several beneficial effects of the UV treatment on the bone healing were observed, which the authors ascribed partly to the decreased carbon contamination levels after treatment. A comparison between autoclaving and *γ*-irradiation indicates that the latter technique has a major advantage: identically prepared and cleaned, but autoclaved, machined titanium implants had 34 at % C contamination [3] whereas in the present study the *γ*-irradiated samples had only 13 at % C. The presently used methods of direct current glow discharge plasma treatment, followed by plasma oxidation or plasma nitriding, and subsequent sterilization with *γ*-irradiation may therefore be of interest for controlled preparation, cleaning, and sterilization of medical implants.

## Figures and Tables

**Table 1:** Results from AES investigation of screw shaped Ti implant surface layers.

Preparation	Chemical composition at % (s.d.)	Thickness (nm)	Microstructure
Machined, reference *γ*-sterilized	Ti: 18 (0.5); O: 59 (1.5); C: 13 (0.8); Ca: 8 (0.7); S: 0.7 (0.2); Si: 1.3 (0.3); Cl: 0.1 (0.1)	*≈*3 nm TiO_2_	Plastically deformed, amorphous metal surface. Non-crystalline oxide (TiO_2_)
Glow discharge cleaned and thermally oxidized, *γ*-sterilized	Ti: 23 (0.8), O: 66 (2); C: 10 (2.7); S: 0.1 (0.1); Si: 0.7 (0.7)	*≈*2 nm TiO_2_	Polycrystalline metal surface. Non-crystalline oxide (TiO_2_)
Glow discharge cleaned and nitrided *γ*-sterilized	Ti: 20 (0.4), N: 59 (1.1); O: 12 (0.8); C: 8 (1.1); Si: 0.6 (0.7);	*≈*3 nm TiN	Polycrystalline metal surface. Non-crystalline nitride (TiN)
H_2_O_2_ incubated 10 mM, 40 h, 8°C	Ti: 17 (3.4), O: 56 (3.2); C: 20 (2.7); Ca: 1.3 (0.3); S: 0.5 (0.2); Si: 0.2 (0.6); Cl: 0.1 (0.1); P: 0.2 (0.3); B: 0.2 (0.5); Na: 2.4 (2.3); K: 0.4 (1.1)	*≈*3–7 nm TiO_2_	Plastically deformed, amorphous metal surface. Noncrystalline oxide (TiO_2_)

**Table 2:** Results from AFM (surface roughness (*R*_rms_), surface area enlargement (*A*_diff_)), and optical profilometer (*R*_*a*_) measurements.

Preparation	*R*_rms_ (nm), mean (s.d.)	*A*_diff_ %, mean (s.d.)	*R*_*a*_ value, (*µ*m)
Machined, reference, *γ*-sterilized	26.3 (17.6)	13.1 (8.96)	0.6
Glow discharge cleaned and thermally oxidized, *γ*-sterilized	10.2 (4.45)	0.78 (0.49)	0.4
Glow discharge cleaned and nitrided, *γ*-sterilized	25.2 (11.1)	8.63 (6.88)	0.6
H_2_O_2_ treated, 10 mM, 40 h, 8°C	25.6 (11.2)	20.5 (5.39)	0.7
